# Use of advanced PET-volume metrics predicts risk of local recurrence and overall survival in anal cancer

**DOI:** 10.1371/journal.pone.0246535

**Published:** 2021-02-04

**Authors:** Matthew S. Susko, Ann A. Lazar, Chia-Ching Jackie Wang, Katherine Van Loon, Mary Feng, Tom A. Hope, Spencer Behr, Mekhail Anwar

**Affiliations:** 1 Department of Radiation Oncology, University of California, San Francisco, California, United States of America; 2 Helen Diller Family Comprehensive Cancer Center, University of California, San Francisco, California, United States of America; 3 Zuckerberg San Francisco General Hospital–Medical Oncology, University of California, San Francisco, California, United States of America; 4 Department of Radiology and Biomedical Imaging, University of California, San Francisco, California, United States of America; Ente Ospedaliero Cantonale, SWITZERLAND

## Abstract

**Objective:**

Anal cancer is an uncommon malignancy with the primary treatment for localized disease being concurrent radiation and chemotherapy. Pre-treatment PET/CT is useful for target delineation, with minimal exploration of its use in prognostication. In the post-treatment setting there is growing evidence for advanced PET metrics in assessment of treatment response, and early identification of recurrence essential for successful salvage, however this data is limited to small series.

**Methods:**

Patient with non-metastatic anal cancer from a single institution were retrospectively reviewed for receipt of pre- and post-treatment PET/CTs. PET data was co-registered with radiation therapy planning CT scans for precise longitudinal assessment of advanced PET metrics including SUV_max_, metabolic tumor volume (MTV), and total lesion glycolysis (TLG), for assessment with treatment outcomes. Treatment outcomes included local recurrence (LR), progression free survival (PFS), and overall survival (OS), as defined from the completed radiation therapy to the time of the event. Cox proportional hazard modeling with inverse probability weighting (IPW) using the propensity score based on age, BMI, T-stage, and radiation therapy dose were utilized for assessment of these metrics.

**Results:**

From 2008 to 2017 there were 72 patients who had pre-treatment PET/CT, 61 (85%) had a single follow up PET/CT, and 35 (49%) had two follow up PET/CTs. The median clinical follow-up time was 25 months (IQR: 13–52) with a median imaging follow up time of 16 months (IQR: 7–29). On pre-treatment PET/CT higher MTV_2.5_ and TLG were significantly associated with higher risk of local recurrence (HR 1.11, 95% CI: 1.06–1.16, p<0.001; and HR 1.12, 95% CI: 1.05–1.19, p<0.001), and worse PFS (HR 1.09, 95% CI: 1.04–1.13, p<0.001; and HR 1.09, 95% CI: 1.03–1.12, p = 0.003) and OS (HR 1.09, 95% CI: 1.04–1.16, p = 0.001; and HR 1.11, 95% CI: 1.04–1.20, p = 0.004). IPW-adjusted pre-treatment PET/CT showed higher MTV_2.5_ (HR 1.09, 95% CI: 1.02–1.17, p = 0.012) and TLG (HR 1.10, 95% CI: 1.00–1.20, p = 0.048) were significantly associated with worse PFS, and post-treatment MTV_2.5_ was borderline significant (HR 1.16, 95% CI: 1.00–1.35, p = 0.052).

**Conclusion:**

Advanced PET metrics, including higher MTV_2.5_ and TLG, in the pre-treatment and post-treatment setting are significantly associated with elevated rates of local recurrence, and worse PFS and OS. This adds to the growing body of literature that PET/CT for patient with ASCC should be considered for prognostication, and additionally is a useful tool for consideration of early salvage or clinical trial of adjuvant therapies.

## Introduction

Anal squamous cell carcinoma (ASCC) is an uncommon malignancy with an estimated 8300 new diagnoses and 1280 deaths in the United States in 2019 [1]. HIV infection is a well-known risk factor for ASCC in men; however, the contemporary rise of ASCC in women is less well understood [2–5]. The primary treatment for non-metastatic ASCC involves radiation and concurrent chemotherapy, consisting of 5-fluorouracil and mitomycin-C, with surgery reserved as a salvage option [6–8]. Modern radiation therapy techniques provide good rates of local control with acceptable rates of toxicity [9], and approximate 5-year disease free survival rates of 60–70% [10]. Patients with large tumors (T3/4) or node positive disease are at highest risk for post-treatment failure.

Initial pretreatment staging using FDG-18 positron emission tomography (PET/CT) has been shown to aid in the characterization of T-stage, pathologic lymph nodes, and distant metastatic disease [11]. PET/CT additionally allows for improved target delineation in radiation treatment planning, as well as alteration of treatment intention a small percentage of patients [12–14]. Aside from solely improving treatment planning, a higher maximum FDG avidity has been associated with poorer rates of disease free survival and increased risk of nodal disease [15].

While surveillance of patients following definitive chemoradiation has commonly been done with anoscopy and CT imaging, mounting evidence supports the use of PET/CT in post-treatment evaluation [16,17]. It has been previously been reported that a complete metabolic response (CMR) on PET/CT portends a more favorable prognosis after treatment for ASCC [18–20]. However, further analysis and investigation of the radiographic features of treatment response, including metabolic tumor volume above standard uptake value (SUV) of 2.5 (MTV_2.5_), total lesion glycolysis (TLG), and SUV_max_ are needed to identify patients at risk for treatment failure. This analysis was performed to evaluate whether radiographic features acquired for pre-treatment prognostication and treatment planning predict for post-treatment response to therapy.

## Methods

### Study design and patient characteristics

In this institutional review board approved study, patients who underwent chemoradiotherapy for ASCC with curative intent between 2005–2018 at the University of California, San Francisco (UCSF) Helen Diller Family Comprehensive Cancer Center was performed. This data was accessed between January 21^st^ 2019 to May 5^th^ 2019. Patients included in this analysis were those who initiated definitive treatment with radiation therapy after pathologic confirmation of diagnosis, and were treated with concurrent 5-FU and Mitomycin-C. For inclusion, patients must have undergone a staging FDG-18 PET/CT prior to radiation planning and confirmed to have no sites of distant metastatic disease at presentation. Baseline patient characteristics including age at diagnosis, gender, HIV status, and staging information were collected for analysis. The institutional review board waived the need for patient consent as this study was deemed minimal risk.

### PET acquisition, treatment, and follow up

Pre-treatment PET/CT was used for delineation of the target volumes and nodal regions at risk and evaluation of post treatment response. Both intensity-modulated radiotherapy (IMRT) and 3D-conformal radiotherapy (3D-CRT) were used for treatment, reflecting evolving practices during the study period. CT simulation was performed for each patient allowing for target and organ at risk (OAR) delineation during treatment planning. Treatment dose and fractionation was at the discretion of the treating physician. During treatment, patients were seen by a radiation oncologist weekly for assessment of toxicity and evaluation for the need for treatment breaks. All patients were treated with concurrent 5-FU and mitomycin-C according to the Nigro regimen [21], with dose reductions at the discretion of the treating medical oncologist.

Post-treatment follow-up was performed by a medical oncologist, radiation oncologist and/or through the specialized Anal Neoplasia Clinic. Patients were assessed per NCCN guidelines for ongoing toxicity from treatment, disease status, and need for ongoing management or treatment. Follow up FDG PET/CT was planned for between 2–6 months from the end of radiation therapy for evaluation of residual or recurrent disease, though specific timing was up to the treating physician. Additional patient data collected include date of last follow up, date of last imaging, dates of follow up PET/CTs, and date of last cross-sectional abdominal and pelvic imaging.

### FDG PET/CT imaging analysis

Pre-treatment and post-treatment FDG PET/CTs were exported from the institutional PACS to MIM (MIM Software, Cleveland OH) for alignment with the CT simulation performed at the time of treatment planning. The CT series of each of the FDG PET/CT was co-registered and fused to the planning CT with the primary site of alignment being the bony structures of the pelvis (sacrum, ilium, ischium, and pubis) using a rigid registration system. Subsequently the PET images, which had been previously registered to the CT series of the PET/CT, were aligned with the planning CT. Given the variability of patient positioning for each imaging session, the PET was then manually adjusted for alignment on the planning CT with the priority of overlapping the tumor volume. Radiation therapy volumes of interest (VOIs) were simplified to include the gross tumor volumes (GTV), clinical tumor volumes (CTV), and planning target volumes (PTVs).

Within MIM, exported contours were then modified to only include the hypermetabolic gross tumor volume within the anal canal, rectum, and nodal regions. The SUV_max_ was defined as the voxel with the highest quantified SUV avidity. MTV_2.5_ was defined as the volume of the gross tumor with SUV greater than 2.5, as has been utilized in prior series [17,22,23]. TLG was defined as the MTV_2.5_ multiplied by the SUV_mean_ of that volume. These characteristics were collected for the pre-treatment PET/CT as well as the post-treatment PET/CT when available. Due to the variability in bladder filling and patient positioning, individual modifications were made to the volumes prior to quantification to avoid overestimation by inclusion of the bladder volume.

### Statistical analysis

Evaluation of discrete categorical variables was performed with either the χ2 or Fisher’s exact test, with comparison of continuous variables using the Wilcoxon Rank-Sum test. Paired comparisons (post vs. pre) were performed using paired t-tests for outcomes measured on the continuum. We also evaluated log transformation of the continuous variables, and since the results were similar, we reported the results based on the untransformed data. For analysis of survival, local recurrence was defined as the time from last radiation treatment to locally recurrent disease, based on pathologic sampling or imaging resulting salvage therapy, or last follow up; progression free survival (PFS) was defined as the time from last radiation treatment to local or distant recurrence of disease, death, or last follow up. Overall survival (OS) was defined as the time elapsed from the date of the last radiation treatment to date of death or last follow up. Univariate Cox proportional hazard model was performed to assess which clinical and PET characteristics were associated with local recurrence, PFS, and OS. Assessment of time-varying covariates were not possible due to limited events (OS: 13 deaths; Local Recurrence: 14 events; PFS: 22 events). Given concern for a limited number of events, only PFS was analyzed using IPW using the propensity score. The propensity score included age (cubic), body mass index (BMI), radiation therapy dose fractions and T stage [24–26]. Robust variance estimation was used to account for the sample weights. Proportional hazards were assessed using an interaction between time and primary covariate, and the p-value for all models were less than 0.05 indicating assumptions met. Hazard ratio (HR) and confidence intervals were presented to display the magnitude of association and degree of uncertainty, with two-sided p-value less than 0.05 considered statistically significant. All analyses were performed in SAS version 9.4.

## Results

A total of 72 patients were identified who initiated definitive treatment for ASCC from 2005–2018 and underwent a pre-treatment PET/CT scan. There were 47 (65%) men and 25 (35%) women with a median age at time of treatment of 57 years (IQR: 50–66). Additional baseline and disease characteristics found on Table 1. The median clinical follow-up time was 25 months (IQR: 13–52) with a median imaging follow up time of 16 months (IQR: 7–29). The median time from completion of treatment until the first post-treatment PET/CT was 3 months (IQR: 1–6), and 7 months (IQR: 6–15) for the second post-treatment PET/CT.

**Table 1:** Patient and treatment characteristics.

Total Patients (n = 72)	n (%)
Median Age (IQR) in years	57 (50–66)
Median BMI (IQR)	25 (22–30)
Gender (%)	
Male	47 (65)
Female	25 (35)
T Stage (%)	
T1	15 (22)
T2	35 (49)
T3	19 (26)
T4	2 (3)
N Stage (%)	
Negative	39 (54)
Positive	33 (46)
HIV-status (%)	
Negative	38 (53)
Positive	34 (47)
Median Radiation Dose in Gy (IQR)	55.8 (54–56)
Median Fractions (IQR)	30 (28–31)
Patient with PET/CT (%)	
Pre-Treatment	72 (100)
Post-Treatment #1	61 (85)
Post-Treatment #2	35 (49)

Within this cohort of patients, 34 (47%) were HIV-positive at the time of treatment initiation. There was no significant association found between HIV status and T-stage (p = 0.56), N-stage (p = 0.93), or the total number of PET/CTs performed per patient (p = 0.69). HIV-positive patients were found to be significantly younger (<0.01) and were predominantly male (p<0.01), as compared to HIV-negative patients. Notably, there were no statistically significant differences between pre-treatment PET metrics of SUV_max_ (p = 0.98), MTV_2.5_ (p = 0.36), or TLG (p = 0.38) between HIV-positive and HIV-negative patients.

**Fig 1:**
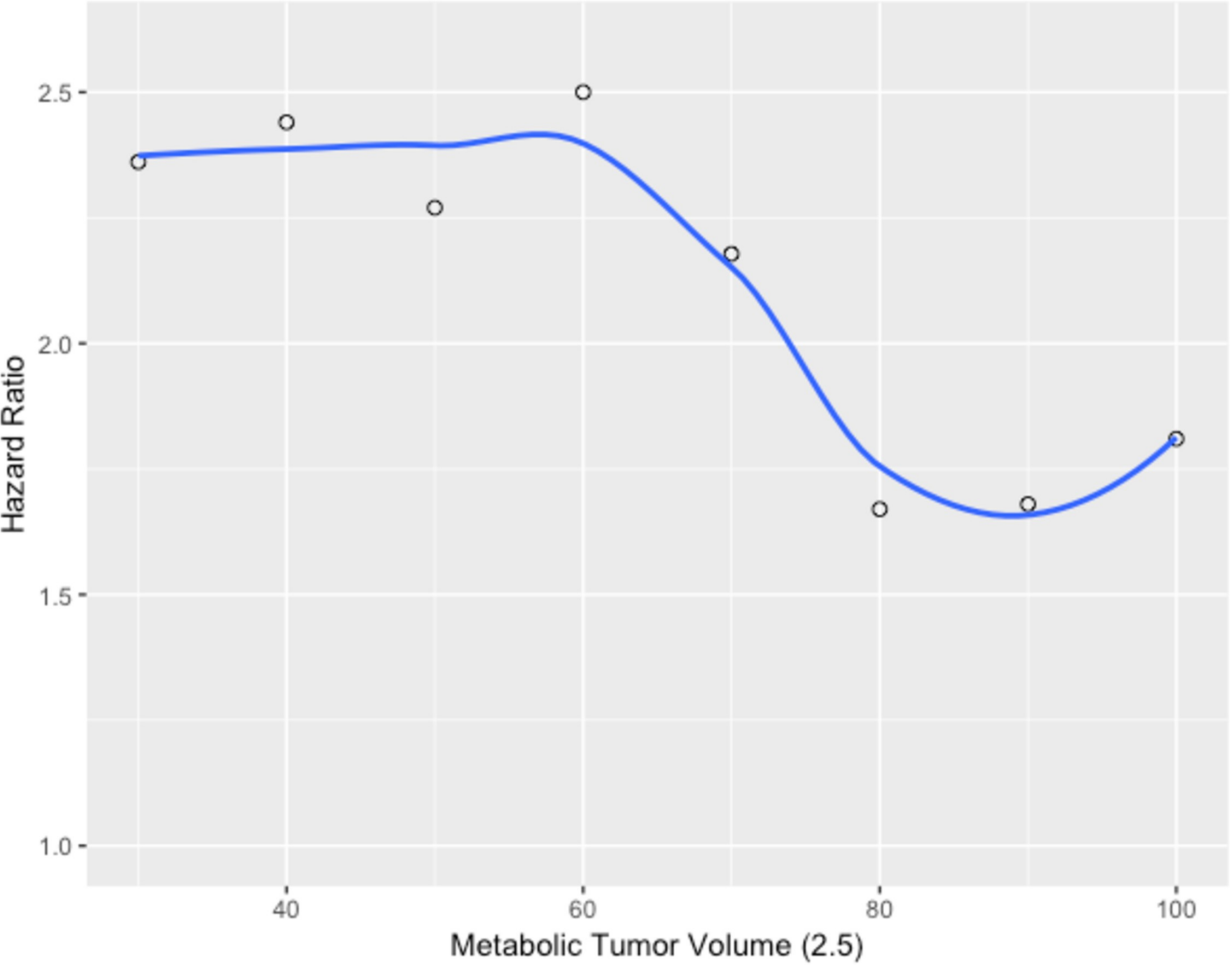
Cox proportional hazard ratio (HR) PFS model of deciles of MTV_2.5_ demonstrating a transition in risk above an MTV_2.5_ threshold of 70 cc. A higher HR denotes a smaller proportion of failures below the MTV_2.5_ threshold.

Of the 72 patients, 61 (85%) had a single follow up PET/CT available for analysis, and 35 (49%) had two follow up PET/CTs available. PET characteristics can be found on Table 2 for the pre-treatment, first post-treatment, and second post-treatment PET/CTs. On initial PET/CT the median SUV_max_ was 11.25 (IQR: 7.41–13.33), the MTV_2.5_ was 22.48 cc (IQR: 10.81–61.45), and the TLG was 94.71 (IQR: 43.14–413.28). When stratified by T stage (T1, T2, and T3/4) the median MTV_2.5_ on the pre-treatment PET/CT was 8.45 cc (IQR: 4.79–14.70), 20.01 cc (IQR: 11.35–46.16), and 79.78 cc (IQR: 26.10–161.34), respectively. Fig 1 demonstrates dichotomized Cox proportional hazard ratios per decile of MTV_2.5_ revealing a transition in HR from above 2.0 at a MTV_2.5_ threshold of 70 cc. Using this cutoff, there were 55 patients with MTV_2.5_ less than 70 cc, with 5 resultant local failures (9.1%) and 17 patients above this threshold with 9 local failures (52.9%).

**Table 2:** PET characteristics.

	Median	IQR
Pre-Treatment		
SUV_max_	11.25	7.41–13.33
MTV_2.5_	22.48	10.81–61.45
TLG	94.71	43.14–413.28
Post-Treatment #1		
SUV_max_	3.98	3.31–5.25
MTV_2.5_	4.67	2.41–12.58
TLG	13.61	6.34–40.69
Post-Treatment #2		
SUV_max_	4.04	3.46–4.38
MTV_2.5_	7.54	2.34–17.72
TLG	21.73	6.45–45.56

**Fig 2:**
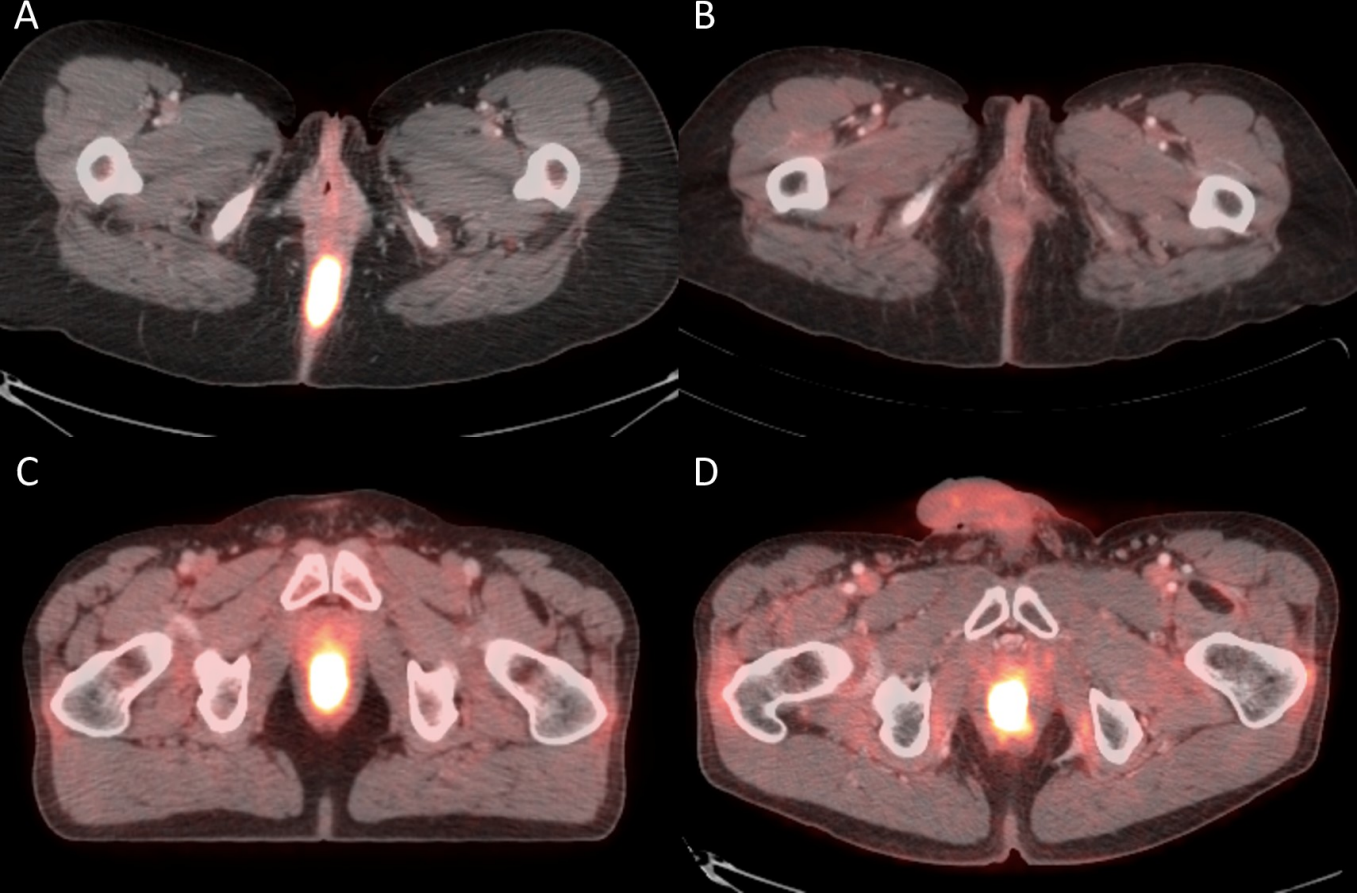
Pre and post-treatment PET/CTs for a patient without (patient 1) and with (patient 2) local recurrence A) Pre-treatment PET/CT of patient 1 with MTV_2.5_ of 49.65cc (B) Post-treatment PET/CT of patient 1 with MTV_2.5_ of 6.21cc C) Pre-treatment PET/CT of patient 2 with MTV_2.5_ of 41.44cc (D) Post-treatment PET/CT of patient 2 with MTV_2.5_ of 21.52cc.

The median percentage decrease in SUV_max_, MTV_2.5_, and TLG from the pre-treatment PET/CT to the initial post treatment PET/CT was 57% (IQR: 36–71), 71% (IQR: 36–89), and 79% (IQR: 60–95) respectively. These values remained stable when comparing the pre-treatment PET/CT metrics to the second post-treatment PET/CT. Fig 2 demonstrates a pre and post-treatment images for a patient without local recurrence (A&B) and for a patient who developed a local recurrence (C&D).

Results of a univariate analysis of the pre-treatment, initial post-treatment, and second post-treatment PET/CT are presented in Table 3. On pre-treatment PET/CT higher values for MTV_2.5_ and TLG were significantly associated with higher risk of local recurrence (HR 1.11, 95% CI: 1.06–1.16, p<0.001; and HR 1.12, 95% CI: 1.05–1.19, p<0.001), and worse PFS (HR 1.09, 95% CI: 1.04–1.13, p<0.001; and HR 1.09, 95% CI: 1.03–1.12, p = 0.003) and OS (HR 1.09, 95% CI: 1.04–1.16, p = 0.001; and HR 1.11, 95% CI: 1.04–1.20, p = 0.004). SUV_max_ on pre-treatment PET/CT was not significantly associated with LR, PFS or OS as demonstrated on Table 3. On initial post-treatment PET/CT MTV_2.5_ remained significantly associated with higher risk of local recurrence, and worse PFS and OS. Additionally, higher SUV_max_ was only significantly associated with risk of local recurrence and worse PFS, but not OS. TLG remained significantly associated with risk(?) of LR and PFS, but was only approaching significance (p = 0.063) for OS. Analysis of the relative change (%) between pre-treatment and post-treatment PET/CT metrics did not reveal any significant differences for local recurrence, PFS, or OS.

**Table 3:** Univariate analysis for LR, PFS, OS*.

	Local Recurrence			PFS			OS		
	HR	95% CI	p-value	HR	95% CI	p-value	HR	95% CI	p-value
Pre-Treatment									
SUV_max_	1.05	0.98–1.12	0.20	1.03	0.97–1.09	0.34	1.02	0.94–1.10	0.63
MTV_2.5_	1.11	1.06–1.16	<0.001	1.09	1.04–1.13	<0.001	1.09	1.04–1.16	0.001
TLG	1.12	1.05–1.19	<0.001	1.09	1.03–1.20	0.003	1.11	1.04–1.20	0.004
Post-Treatment #1									
SUV_max_	1.22	1.07–1.39	0.003	1.18	1.04–1.35	0.011	1.08	0.82–1.41	0.59
MTV_2.5_	1.21	1.10–1.33	<0.001	1.18	1.07–1.29	<0.001	1.17	1.02–1.35	0.026
TLG	1.44	1.17–1.76	<0.001	1.37	1.12–1.67	0.002	1.33	0.98–1.80	0.063
Post-Treatment #2									
SUV_max_	1.32	1.05–1.67	0.019	1.20	0.98–1.48	0.082			
MTV_2.5_	1.46	0.89–2.40	0.14	1.18	0.72–1.94	0.51			
TLG	4.20	0.89–19.7	0.069	2.10	0.48–9.30	0.32			

* HR for MTV_2.5_ are expressed per 10 cc, HR for TLG are expressed per 100 units.

To account for factors which were significantly associated with PFS, IPW was performed. Prior to analysis, the decision was made to restrict this modeling to PFS, given the small number of events in the local recurrence and OS models. Results are presented in Table 4, demonstrating that higher MTV_2.5_ and TLG on pre-treatment PET/CT were significantly associated with worse PFS when controlling for age, BMI, T-stage, and radiation therapy dose. On initial post-treatment PET/CT, MTV_2.5_ remained close to statistical significance (HR: 1.16, 95% CI: 1.02–1.17, p = 0.052). Due to the decreased number of patients with two post-treatment PET/CT scans, this analysis was not able to be performed for the second post-treatment PET/CT.

**Table 4:** Inverse probability model PFS*.

	HR	95% CI	p-value
Pre-Treatment			
SUV_max_	0.99	0.88–1.10	0.84
MTV_2.5_	1.09	1.02–1.17	0.012
TLG	1.10	1.00–1.20	0.048
Post-Treatment #1			
SUV_max_	1.08	0.81–0.41	0.59
MTV_2.5_	1.16	1.00–1.35	0.052
TLG	1.30	0.99–1.60	0.10

* HR for MTV_2.5_ are expressed per 10 cc, HR for TLG are expressed per 100 units.

## Discussion

This study demonstrated that advanced PET/CT metrics, including MTV_2.5_ and TLG, on pre-treatment imaging are prognostic for risk of local recurrence, PFS, and OS. Additionally, in the 2–6 months after treatment, MTV_2.5_ derived via post-treatment PET/CT continues to have significant association with these outcome metrics. When inverse probability modeling is performed for PFS to control for T-stage, age, BMI, and radiation treatment dose, MTV_2.5_ continued to be significant on pre-treatment PET/CT and approached significance (p = 0.052) on initial post-treatment PET/CT. This data supports the routine use of pre-treatment and post-treatment PET/CT in patients with ASCC.

FDG PET at the time of diagnosis of ASCC is beneficial for initial staging and use in target delineation of the primary in radiation therapy treatment planning [12,13,27]. Additionally, lymph node evaluation with PET/CT has been shown to upstage patients and change management with reasonable sensitivity [28,29]. The rate of PET positivity for nodal staging with PET/CT for HIV-positive patients has been demonstrated to be increased over HIV-negative patients; however, the magnitude of this difference is unknown [28,30]. Further evaluation of the difference between a larger cohort of HIV-positive and HIV-negative patients, inclusive of the entire cohort of patients in this study has previously been reported [31]. In the current study, we found no differences amongst pre-treatment PET/CT metrics and HIV status, however those same metrics were predictive of outcomes for local response, PFS, and OS. The most robust of these metrics appears to be MTV_2.5,_ which maintains significance for all outcomes and could be beneficial as a means of further delineating radiation therapy planning volumes for consideration of dose escalation.

Overall, the role of FDG PET/CT in prognosis has been examined in a limited number of studies. One study has demonstrated that a higher pretreatment FDG PET/CT SUV_max_ is associated with worse disease-free survival, and increased rate of lymph node involvement, independent of tumor size [15]. Conversely, a recent prospective evaluation of PET/CT in ASCC did not reveal an association between pre-treatment PET metrics, including MTV and TLG, and any recurrence. The latter study was significantly limited in sample size with only 19 patients accrued and 16 months of follow up, likely limiting its ability to detect a significant association. The data of the current study is more robust, with 72 patients undergoing pre-treatment PET/CT, and the notable demonstration that MTV_2.5_ and TLG were associated with risk for local recurrence, as well as worse PFS and OS. When controlled for T-stage and other clinical factors these pre-treatment metrics (MTV_2.5_ and TLG) remained significant, something novel that the current study has demonstrated. These results are supported by an analysis of multiple studies which evaluated the use of PET/CT in the treatment of ASCC [32]. Additionally, a novel finding of this study was that an MTV_2.5_ threshold of 70 cc appears to differentiate a low and high-risk cohort for local recurrence.

The use of post-treatment PET/CT follow-up for ASCC is not required per NCCN; however, there is mounting evidence of its effectiveness in the early detection of recurrence and residual disease for post-treatment prognostication. Typically, clinical follow up involves digital rectal exam (DRE), anoscopy, and contrast-enhanced CT in patients with high risk disease, with those found to have incomplete response to radiation therapy having a worse overall prognosis [33]. This influenced the patterns of care within the current study, such that patients with incomplete response or higher risk disease, were more likely to undergo multiple PET/CTs for disease evaluation. However, given the heterogeneity of patient with ASCC, further characterization of this could not be explored but should be considered for future studies.

The use of PET/CT in the post-treatment setting has demonstrated that complete-metabolic response (CMR) is predictive of improved PFS and cause-specific mortality (CSS) with a 2-year CSS of 100% and 96% in patients with CMR and 59% and 39% in patients without CMR [18,20]. Even in the setting of mid-treatment PET/CT, MTV_2.5_ and TLG have been shown to correlate with increased risk for local recurrence [17]. Given these results, strong consideration should be given for recommendation of pre and post-treatment PET/CT in prognostication and assessment of treatment response for ASCC patients. This information could aid in the decision to provide early salvage therapy, and if performed during mid-treatment consideration of treatment adaptation and dose-escalation if there is concern for high-risk disease or lack of treatment response.

This study was limited by its retrospective nature and the use of both 3D-CRT and IMRT in the treatment of patients with ASCC during the study period. While retrospective, the patients were treated in a consistent manner at a single institution allowing for minimal variability amongst patient treatment plans. While the differences in technique between 3DCRT and IMRT could lead to differences in outcomes, there were no differences in the radiation doses patients were treated with, and 3D-CRT likely utilized larger margins compared to IMRT, possibly leading to increased toxicity but not worse local control or treatment response. One additional consideration is that the timing of the post-treatment PET/CTs was not standardized, and therefore this study is not able to answer the question of the ideal timing for assessment of treatment response. Also, the lack of significance of the relative change of the pre and post-treatment PET metrics in predicting outcomes may result in different results if other techniques and technologies are used. Overall, these results are meant to elucidate an initial validation of the use of PET-volume metrics in anal cancer and further prospective studies will need to be performed to further support these results.

## Conclusion

Advanced PET/CT metrics, inclusive of MTV_2.5_ and TLG are predictive of local recurrence, PFS, and OS in the pre-treatment setting, and remain significant on post-treatment imaging. On multivariate modeling for PFS, which has never previously been reported for ASCC, significant association was demonstrated for MTV_2.5_ and TLG when controlling for T-stage and treatment variables. PET/CT should be considered a useful tool for both pre-treatment and post-treatment prognostication and assessment of treatment response in ASCC, with the potential use of MTV_2.5_ of 70 cc as a risk stratification for high and low risk of failure. Those without adequate reduction of MTV_2.5_ or TLG on post-treatment imaging should be considered for early salvage, close surveillance, or consideration of a clinical trial of adjuvant treatment.
