# Supplementary material for: Use of advanced PET-volume metrics predicts risk of local recurrence and overall survival in anal cancer
Source: PLoS One. 2021 Feb 4;16(2):e0246535. doi: 10.1371/journal.pone.0246535 (PMC7861457; doi:10.1371/journal.pone.0246535)
Supplement: Attachment — Submitted filename: ASCC_PO_Response.docx [file pone.0246535.s001.docx]

Dear Dr. Treglia and the reviewers at PLOS ONE,

Thank you for your time and thoughts regarding the review of our manuscript, “Use of advanced PET-volume metrics predicts risk of local recurrence and overall survival in anal cancer.” We have responded to your comments and revised our manuscript appropriately, with the changes outlined below.

Comments:

1. Please ensure that your manuscript meets PLOS ONE's style requirements

The manuscript has been revised to meet the style requirements

2. Please provide additional details regarding participant consent.

We have added, “The institutional review board waived the need for patient consent as this study was deemed minimal risk.” to the methods section.

3. Thank you for stating that you included "patients who underwent chemoradiotherapy for ASCC with curative intent between 2008-2017". Please also state the date(s) on which you accessed the databases or records to obtain your data.

We have added, “This data was accessed between January 21st 2019 to May 5th 2019.” to the manuscript.

4. We note that you have indicated that data from this study are available upon request. PLOS only allows data to be available upon request if there are legal or ethical restrictions on sharing data publicly.

Data cannot be shared publicly because of protected health information. Data are available from the UCSF Institutional Data Access / Ethics Committee (contact via irb@ucsf.edu) for researchers who meet the criteria for access to confidential data.

5. Please amend your list of authors on the manuscript to ensure that each author is linked to an affiliation.

We have added a link to each authors affiliation per the PLOS ONE style guide.

Reviewer #1

1. There is a systematic review and meta-analysis on this subject ([https://doi.org/10.1155/2018/9760492](https://urldefense.proofpoint.com/v2/url?u=https-3A__doi.org_10.1155_2018_9760492&d=DwQGaQ&c=iORugZls2LlYyCAZRB3XLg&r=zzOQc9KSyyIo_1Lnr98XJpRyHePCJijdAmqS9_U1Dac&m=4vzJ0CknM8pQWYXA04NMl6QpE5IaDMwbhdYG57_OIlw&s=Fq2R-h8BxUwL68xrWDouxmzS4_XtVwXzfD2FDaBlPNA&e=)). The authors may discuss the difference or similarity of their results to the this systematic review.

We have added the statement, “These results are supported by an analysis of multiple studies which evaluated the use of PET/CT in the treatment of ASCC.” with a citation to the referenced analysis.

Reviewer #2

1) Due to the retrospective design of the paper, it is expected that the 35 patients performing 2 follow-up PET-CTs were in a more advanced T and N stage of disease than the ones performing only one follow-up PET-CT: it is possible that a second follow-up PET-CT was preferred in patients who showed a higher disease burden at first follow-up PET-CT or even at baseline one. It is evident from Table 2 that PET metrics on second follow-up PET-CT are slightly higher than on first follow-up PET-CT: is it justified by the inclusion of patients with a higher burden of disease and consequently worse PET metrics on baseline and/or first follow-up PET-CT, or not? Or these of PET metrics are justified by initial lack of response? Please explain.

We have added, “This influenced the patterns of care within the current study, such that patients with incomplete response or higher risk disease, were more likely to undergo multiple PET/CTs for disease evaluation. However, given the heterogeneity of patient with ASCC, further characterization of this could not be explored but should be considered for future studies.” to the manuscript. While this is an important factor to consider the nature of treatment of ASCC is such that patient with lower risk disease are more likely to have good response to treatment and thus not require further metabolic imaging. Our study reflects the practicalities of this and the needs to have increased imaging for higher risk patients.

2) Has a sub-analysis in terms of outcome for HIV-positive and HIV-negative patients been performed separately? Indeed, it should be useful to know whether PET metrics are the only predictive factors for local recurrence or PFS regardless of HIV infection status (in this case, predictive role has to be maintained at separated evaluation), althogh it has been showed that PET metrics are not quite diffeent between the two groups. HIV status was not included in IPW factors.

We have added, “Further evaluation of the difference between a larger cohort of HIV-positive and HIV-negative patients, inclusive of the entire cohort of patients in this study has previously been reported.” with a citation to this analysis.

3) It is interesting that relative baseline and post-treatment % changes of PET metrics did not reveal anly significant differences in terms of local recurrence, PFS or OS, while absolute values did it: in terms of repeatibility of results using different technical equipment, this could be a limitation; this aspect should be underlined also in the discussion section.

We have added, “Also, the lack of significance of the relative change of the pre and post-treatment PET metrics in predicting outcomes may result in different results if other techniques and technologies are used.” While the lack of significance of the relative change in metrics is important to note, this manuscript establishes absolute cutoff values and a strict methodology to evaluate them that should allow others to reproduce the results effectively.

Please contact us if you have further questions or concerns. We look forward to working with you toward publication of this manuscript.

Sincerely,

Matt Susko
