# Supplementary material for: Use of advanced PET-volume metrics predicts risk of local recurrence and overall survival in anal cancer
Source: PLoS One. 2021 Feb 4;16(2):e0246535. doi: 10.1371/journal.pone.0246535 (PMC7861457; doi:10.1371/journal.pone.0246535)
Supplement: Peer review history

# Peer review: Decision Letter 0

**DOI:** 10.1371/journal.pone.0246535.r001

**Authors:** Giorgio Treglia

16 Dec 2020

PONE-D-20-35492

Use of advanced PET-volume metrics predicts risk of local recurrence and overall survival in anal cancer

PLOS ONE

Dear Dr. Susko,

Thank you for submitting your manuscript to PLOS ONE. After careful consideration, we feel that it has merit but does not fully meet PLOS ONE’s publication criteria as it currently stands. Therefore, we invite you to submit a revised version of the manuscript that addresses the points raised during the review process.

Please submit your revised manuscript by Jan 22 2021 11:59PM. If you will need more time than this to complete your revisions, please reply to this message or contact the journal office at plosone@plos.org. When you're ready to submit your revision, log on to https://www.editorialmanager.com/pone/ and select the 'Submissions Needing Revision' folder to locate your manuscript file.

Please include the following items when submitting your revised manuscript:

- A rebuttal letter that responds to each point raised by the academic editor and reviewer(s). You should upload this letter as a separate file labeled 'Response to Reviewers'.
- A marked-up copy of your manuscript that highlights changes made to the original version. You should upload this as a separate file labeled 'Revised Manuscript with Track Changes'.
- An unmarked version of your revised paper without tracked changes. You should upload this as a separate file labeled 'Manuscript'.

If you would like to make changes to your financial disclosure, please include your updated statement in your cover letter. Guidelines for resubmitting your figure files are available below the reviewer comments at the end of this letter.

If applicable, we recommend that you deposit your laboratory protocols in protocols.io to enhance the reproducibility of your results. Protocols.io assigns your protocol its own identifier (DOI) so that it can be cited independently in the future. For instructions see: http://journals.plos.org/plosone/s/submission-guidelines#loc-laboratory-protocols

We look forward to receiving your revised manuscript.

Kind regards,

Giorgio Treglia, MD, MSc

Academic Editor

PLOS ONE

Journal Requirements:

When submitting your revision, we need you to address these additional requirements.

1. Please ensure that your manuscript meets PLOS ONE's style requirements, including those for file naming. The PLOS ONE style templates can be found at

https://journals.plos.org/plosone/s/file?id=wjVg/PLOSOne_formatting_sample_main_body.pdf and

https://journals.plos.org/plosone/s/file?id=ba62/PLOSOne_formatting_sample_title_authors_affiliations.pdf

2. Please provide additional details regarding participant consent. We note that you state "Patient data was deemed minimal risk and therefore consent was not obtained." Please clarify if the ethics committee specifically waived the need for informed consent for this study.

3. Thank you for stating that you included "patients who underwent chemoradiotherapy for ASCC with curative intent between 2008-2017". Please also state the date(s) on which you accessed the databases or records to obtain your data.

4. We note that you have indicated that data from this study are available upon request. PLOS only allows data to be available upon request if there are legal or ethical restrictions on sharing data publicly. For information on unacceptable data access restrictions, please see http://journals.plos.org/plosone/s/data-availability#loc-unacceptable-data-access-restrictions.

In your revised cover letter, please address the following prompts:

a) If there are ethical or legal restrictions on sharing a de-identified data set, please explain them in detail (e.g., data contain potentially identifying or sensitive patient information) and who has imposed them (e.g., an ethics committee). Please also provide contact information for a data access committee, ethics committee, or other institutional body to which data requests may be sent.

b) If there are no restrictions, please upload the minimal anonymized data set necessary to replicate your study findings as either Supporting Information files or to a stable, public repository and provide us with the relevant URLs, DOIs, or accession numbers. Please see http://www.bmj.com/content/340/bmj.c181.long for guidelines on how to de-identify and prepare clinical data for publication. For a list of acceptable repositories, please see http://journals.plos.org/plosone/s/data-availability#loc-recommended-repositories.

We will update your Data Availability statement on your behalf to reflect the information you provide.

5. Please amend your list of authors on the manuscript to ensure that each author is linked to an affiliation. Authors’ affiliations should reflect the institution where the work was done (if authors moved subsequently, you can also list the new affiliation stating “current affiliation:….” as necessary).

[Note: HTML markup is below. Please do not edit.]

Reviewers' comments:

Reviewer's Responses to Questions

**Comments to the Author**

1. Is the manuscript technically sound, and do the data support the conclusions?

The manuscript must describe a technically sound piece of scientific research with data that supports the conclusions. Experiments must have been conducted rigorously, with appropriate controls, replication, and sample sizes. The conclusions must be drawn appropriately based on the data presented. 

Reviewer #1: Yes

Reviewer #2: Yes

**********

2. Has the statistical analysis been performed appropriately and rigorously? 

Reviewer #1: Yes

Reviewer #2: Yes

**********

3. Have the authors made all data underlying the findings in their manuscript fully available?

The PLOS Data policy requires authors to make all data underlying the findings described in their manuscript fully available without restriction, with rare exception (please refer to the Data Availability Statement in the manuscript PDF file). The data should be provided as part of the manuscript or its supporting information, or deposited to a public repository. For example, in addition to summary statistics, the data points behind means, medians and variance measures should be available. If there are restrictions on publicly sharing data—e.g. participant privacy or use of data from a third party—those must be specified.

Reviewer #1: No

Reviewer #2: Yes

**********

4. Is the manuscript presented in an intelligible fashion and written in standard English?

PLOS ONE does not copyedit accepted manuscripts, so the language in submitted articles must be clear, correct, and unambiguous. Any typographical or grammatical errors should be corrected at revision, so please note any specific errors here.

Reviewer #1: Yes

Reviewer #2: Yes

**********

5. Review Comments to the Author

Please use the space provided to explain your answers to the questions above. You may also include additional comments for the author, including concerns about dual publication, research ethics, or publication ethics. (Please upload your review as an attachment if it exceeds 20,000 characters)

Reviewer #1: Dear editor,

Thank you for giving me the opportunity to review for your prestigious journal.

The manuscript under review is a well written and well performed study on the prognostic significance of FDG PET/CT in anal canal cancer.

The methodology is sound. As anal canal cancer is very rare, studies on this cancer can be very helpful.

My comments can be found below to improve the manuscript.

1- There is a systematic review and meta-analysis on this subject (https://doi.org/10.1155/2018/9760492). The authors may discuss the difference or similarity of their results to the this systematic review.

best wishes,

Ramin Sadeghi ,MD

Reviewer #2: The Authors have retrospectively evaluated the usefulness of advanced PET metrics (SUVmax, MTV and TLG) in predicting the outcome of patients with anal cancer in the absence of distant metastases. They found that higher MTV2.5 and TLG are associated with elevated rates of local recurrence and worse PFS and OS in both pre-treatment and post-treatment settings, therefore making PET-CT a reliable imaging tool in evaluating and following-up patients with localized disease addressed to radiation therapy. Overall the paper is well written, results are clearly written and compared with existing literature in the discussion section.

However some minor concerns should be addressed to give this paper a higher scientific impact:

1) Due to the retrospective design of the paper, it is expected that the 35 patients performing 2 follow-up PET-CTs were in a more advanced T and N stage of disease than the ones performing only one follow-up PET-CT: it is possible that a second follow-up PET-CT was preferred in patients who showed a higher disease burden at first follow-up PET-CT or even at baseline one. It is evident from Table 2 that PET metrics on second follow-up PET-CT are slightly higher than on first follow-up PET-CT: is it justified by the inclusion of patients with a higher burden of disease and consequently worse PET metrics on baseline and/or first follow-up PET-CT, or not? Or these of PET metrics are justified by initial lack of response? Please explain.

2) Has a sub-analysis in terms of outcome for HIV-positive and HIV-negative patients been performed separately? Indeed, it should be useful to know whether PET metrics are the only predictive factors for local recurrence or PFS regardless of HIV infection status (in this case, predictive role has to be maintained at separated evaluation), althogh it has been showed that PET metrics are not quite diffeent between the two groups. HIV status was not included in IPW factors.

3) It is interesting that relative baseline and post-treatment % changes of PET metrics did not reveal anly significant differences in terms of local recurrence, PFS or OS, while absolute values did it: in terms of repeatibility of results using different technical equipment, this could be a limitation; this aspect should be underlined also in the discussion section.

I suggest a terminological correction: in the interoduction section, last paragraph, the Authors use the term "radiographic" features talking about SUV, MTV and TLG: the term "functional" is, in my opinion, more appropriate.

**********

6. PLOS authors have the option to publish the peer review history of their article (what does this mean?). If published, this will include your full peer review and any attached files.

If you choose “no”, your identity will remain anonymous but your review may still be made public.

**Do you want your identity to be public for this peer review?** For information about this choice, including consent withdrawal, please see our Privacy Policy.

Reviewer #1: **Yes: **Ramin Sadeghi, MD

Reviewer #2: No

[NOTE: If reviewer comments were submitted as an attachment file, they will be attached to this email and accessible via the submission site. Please log into your account, locate the manuscript record, and check for the action link "View Attachments". If this link does not appear, there are no attachment files.]

While revising your submission, please upload your figure files to the Preflight Analysis and Conversion Engine (PACE) digital diagnostic tool, https://pacev2.apexcovantage.com/. PACE helps ensure that figures meet PLOS requirements. To use PACE, you must first register as a user. Registration is free. Then, login and navigate to the UPLOAD tab, where you will find detailed instructions on how to use the tool. If you encounter any issues or have any questions when using PACE, please email PLOS at figures@plos.org. Please note that Supporting Information files do not need this step.

# Author comment: Author response to Decision Letter 0

**DOI:** 10.1371/journal.pone.0246535.r002

13 Jan 2021

Dear Dr. Treglia and the reviewers at PLOS ONE,

Thank you for your time and thoughts regarding the review of our manuscript, “Use of advanced PET-volume metrics predicts risk of local recurrence and overall survival in anal cancer.” We have responded to your comments and revised our manuscript appropriately, with the changes outlined below.

Comments:

1. Please ensure that your manuscript meets PLOS ONE's style requirements

The manuscript has been revised to meet the style requirements

2. Please provide additional details regarding participant consent.

We have added, “The institutional review board waived the need for patient consent as this study was deemed minimal risk.” to the methods section. 

3. Thank you for stating that you included "patients who underwent chemoradiotherapy for ASCC with curative intent between 2008-2017". Please also state the date(s) on which you accessed the databases or records to obtain your data.

We have added, “This data was accessed between January 21st 2019 to May 5th 2019.” to the manuscript.

4. We note that you have indicated that data from this study are available upon request. PLOS only allows data to be available upon request if there are legal or ethical restrictions on sharing data publicly.

Data cannot be shared publicly because of protected health information. Data are available from the UCSF Institutional Data Access / Ethics Committee (contact via irb@ucsf.edu) for researchers who meet the criteria for access to confidential data.

5. Please amend your list of authors on the manuscript to ensure that each author is linked to an affiliation. 

We have added a link to each authors affiliation per the PLOS ONE style guide.

Reviewer #1

1. There is a systematic review and meta-analysis on this subject (https://doi.org/10.1155/2018/9760492). The authors may discuss the difference or similarity of their results to the this systematic review.

We have added the statement, “These results are supported by an analysis of multiple studies which evaluated the use of PET/CT in the treatment of ASCC.” with a citation to the referenced analysis.

Reviewer #2

1) Due to the retrospective design of the paper, it is expected that the 35 patients performing 2 follow-up PET-CTs were in a more advanced T and N stage of disease than the ones performing only one follow-up PET-CT: it is possible that a second follow-up PET-CT was preferred in patients who showed a higher disease burden at first follow-up PET-CT or even at baseline one. It is evident from Table 2 that PET metrics on second follow-up PET-CT are slightly higher than on first follow-up PET-CT: is it justified by the inclusion of patients with a higher burden of disease and consequently worse PET metrics on baseline and/or first follow-up PET-CT, or not? Or these of PET metrics are justified by initial lack of response? Please explain.

We have added, “This influenced the patterns of care within the current study, such that patients with incomplete response or higher risk disease, were more likely to undergo multiple PET/CTs for disease evaluation. However, given the heterogeneity of patient with ASCC, further characterization of this could not be explored but should be considered for future studies.” to the manuscript. While this is an important factor to consider the nature of treatment of ASCC is such that patient with lower risk disease are more likely to have good response to treatment and thus not require further metabolic imaging. Our study reflects the practicalities of this and the needs to have increased imaging for higher risk patients.

2) Has a sub-analysis in terms of outcome for HIV-positive and HIV-negative patients been performed separately? Indeed, it should be useful to know whether PET metrics are the only predictive factors for local recurrence or PFS regardless of HIV infection status (in this case, predictive role has to be maintained at separated evaluation), althogh it has been showed that PET metrics are not quite diffeent between the two groups. HIV status was not included in IPW factors.

We have added, “Further evaluation of the difference between a larger cohort of HIV-positive and HIV-negative patients, inclusive of the entire cohort of patients in this study has previously been reported.” with a citation to this analysis.

3) It is interesting that relative baseline and post-treatment % changes of PET metrics did not reveal anly significant differences in terms of local recurrence, PFS or OS, while absolute values did it: in terms of repeatibility of results using different technical equipment, this could be a limitation; this aspect should be underlined also in the discussion section.

We have added, “Also, the lack of significance of the relative change of the pre and post-treatment PET metrics in predicting outcomes may result in different results if other techniques and technologies are used.” While the lack of significance of the relative change in metrics is important to note, this manuscript establishes absolute cutoff values and a strict methodology to evaluate them that should allow others to reproduce the results effectively.

Please contact us if you have further questions or concerns. We look forward to working with you toward publication of this manuscript.

Sincerely,

Matt Susko

Attachment Submitted filename: ASCC_PO_Response.docx Click here for additional data file.

# Peer review: Decision Letter 1

**DOI:** 10.1371/journal.pone.0246535.r003

**Authors:** Giorgio Treglia

21 Jan 2021

Use of advanced PET-volume metrics predicts risk of local recurrence and overall survival in anal cancer

PONE-D-20-35492R1

Dear Dr. Susko,

We’re pleased to inform you that your manuscript has been judged scientifically suitable for publication and will be formally accepted for publication once it meets all outstanding technical requirements.

Within one week, you’ll receive an e-mail detailing the required amendments. When these have been addressed, you’ll receive a formal acceptance letter and your manuscript will be scheduled for publication.

An invoice for payment will follow shortly after the formal acceptance. To ensure an efficient process, please log into Editorial Manager at http://www.editorialmanager.com/pone/, click the 'Update My Information' link at the top of the page, and double check that your user information is up-to-date. If you have any billing related questions, please contact our Author Billing department directly at authorbilling@plos.org.

If your institution or institutions have a press office, please notify them about your upcoming paper to help maximize its impact. If they’ll be preparing press materials, please inform our press team as soon as possible -- no later than 48 hours after receiving the formal acceptance. Your manuscript will remain under strict press embargo until 2 pm Eastern Time on the date of publication. For more information, please contact onepress@plos.org.

Kind regards,

Giorgio Treglia, MD, MSc

Academic Editor

PLOS ONE

Additional Editor Comments (optional):

Reviewers' comments:

Reviewer's Responses to Questions

**Comments to the Author**

1. If the authors have adequately addressed your comments raised in a previous round of review and you feel that this manuscript is now acceptable for publication, you may indicate that here to bypass the “Comments to the Author” section, enter your conflict of interest statement in the “Confidential to Editor” section, and submit your "Accept" recommendation.

Reviewer #1: All comments have been addressed

Reviewer #2: All comments have been addressed

**********

2. Is the manuscript technically sound, and do the data support the conclusions?

The manuscript must describe a technically sound piece of scientific research with data that supports the conclusions. Experiments must have been conducted rigorously, with appropriate controls, replication, and sample sizes. The conclusions must be drawn appropriately based on the data presented. 

Reviewer #1: Yes

Reviewer #2: Yes

**********

3. Has the statistical analysis been performed appropriately and rigorously? 

Reviewer #1: Yes

Reviewer #2: Yes

**********

4. Have the authors made all data underlying the findings in their manuscript fully available?

The PLOS Data policy requires authors to make all data underlying the findings described in their manuscript fully available without restriction, with rare exception (please refer to the Data Availability Statement in the manuscript PDF file). The data should be provided as part of the manuscript or its supporting information, or deposited to a public repository. For example, in addition to summary statistics, the data points behind means, medians and variance measures should be available. If there are restrictions on publicly sharing data—e.g. participant privacy or use of data from a third party—those must be specified.

Reviewer #1: (No Response)

Reviewer #2: Yes

**********

5. Is the manuscript presented in an intelligible fashion and written in standard English?

PLOS ONE does not copyedit accepted manuscripts, so the language in submitted articles must be clear, correct, and unambiguous. Any typographical or grammatical errors should be corrected at revision, so please note any specific errors here.

Reviewer #1: Yes

Reviewer #2: Yes

**********

6. Review Comments to the Author

Please use the space provided to explain your answers to the questions above. You may also include additional comments for the author, including concerns about dual publication, research ethics, or publication ethics. (Please upload your review as an attachment if it exceeds 20,000 characters)

Reviewer #1: (No Response)

Reviewer #2: (No Response)

**********

7. PLOS authors have the option to publish the peer review history of their article (what does this mean?). If published, this will include your full peer review and any attached files.

If you choose “no”, your identity will remain anonymous but your review may still be made public.

**Do you want your identity to be public for this peer review?** For information about this choice, including consent withdrawal, please see our Privacy Policy.

Reviewer #1: **Yes: **Ramin Sadeghi, MD

Reviewer #2: No

# Editor report: Acceptance letter

**DOI:** 10.1371/journal.pone.0246535.r004

**Authors:** Giorgio Treglia

25 Jan 2021

PONE-D-20-35492R1 

Use of advanced PET-volume metrics predicts risk of local recurrence and overall survival in anal cancer 

Dear Dr. Susko:

I'm pleased to inform you that your manuscript has been deemed suitable for publication in PLOS ONE. Congratulations! Your manuscript is now with our production department. 

If your institution or institutions have a press office, please let them know about your upcoming paper now to help maximize its impact. If they'll be preparing press materials, please inform our press team within the next 48 hours. Your manuscript will remain under strict press embargo until 2 pm Eastern Time on the date of publication. For more information please contact onepress@plos.org.

If we can help with anything else, please email us at plosone@plos.org. 

Thank you for submitting your work to PLOS ONE and supporting open access. 

Kind regards, 

PLOS ONE Editorial Office Staff

on behalf of

Dr. Giorgio Treglia 

Academic Editor

PLOS ONE
